# Supplementary material for: α-Synuclein-specific T cell reactivity is associated with preclinical and early Parkinson’s disease
Source: Nat Commun. 2020 Apr 20;11:1875. doi: 10.1038/s41467-020-15626-w (PMC7171193; doi:10.1038/s41467-020-15626-w)
Supplement: Supplementary file 1 — Supplementary Information [file 41467_2020_15626_MOESM1_ESM.pdf]

α-Synuclein-specific T cell reactivity is associated with preclinical and early Parkinson's disease

Lindestam Arlehamn et al.

a

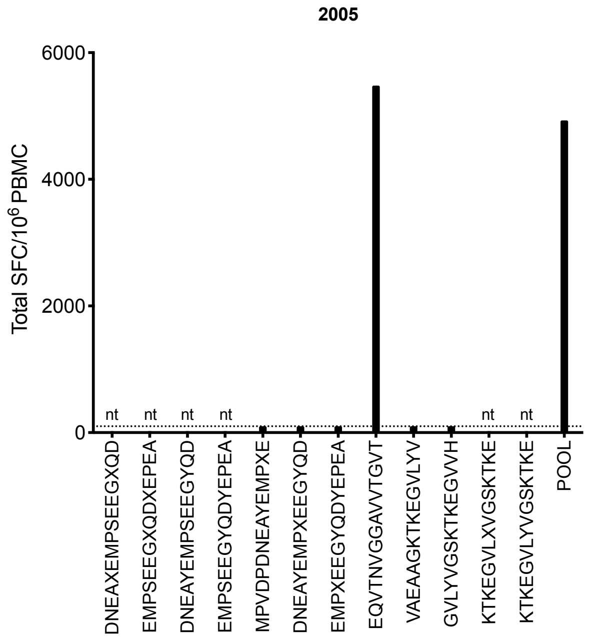

b

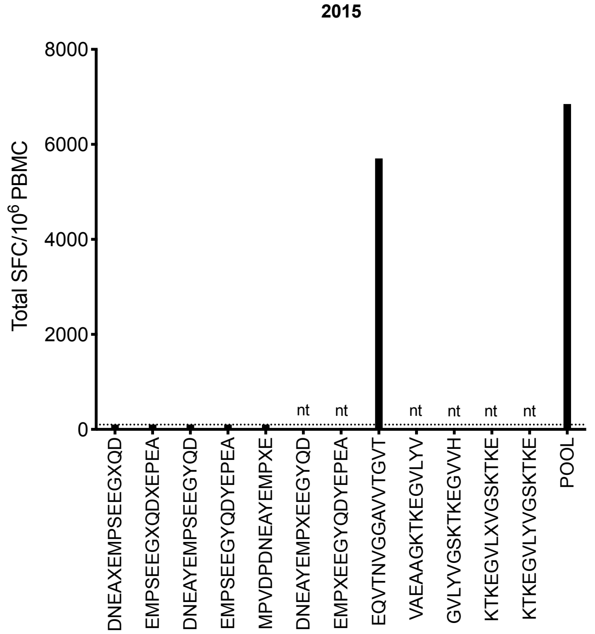

Supplementary Figure 1. **α-syn-specific T cell responses in a longitudinal case study of PD.** Total magnitude of response expressed as total SFC (sum of IFNγ, IL-5, and IL-10 responses) per 10^6 cultured PBMC against a pool of α-syn peptides (furthest to the right) and individual α-syn peptides included in the pool. Due to limited cell numbers, not all peptides were tested, these are indicated by “nt” in the figure. (a) Sample from 2005, (b) sample from 2015.

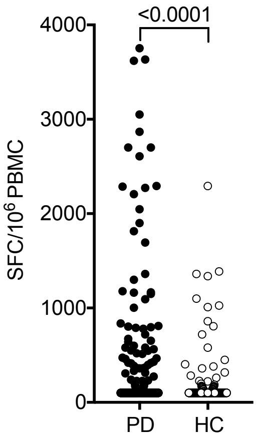

Supplementary Figure 2. Reactivity to α-syn peptides in subjects with PD and HC. Magnitude of responses (sum of IFNγ, IL-5, and IL-10) against α-syn peptides as SFC per 10^6 cultured PBMC. Each point represents a peptide and participant combination. Closed circles, patients with PD (n=514 peptide and participant combinations); open circles, control (n=603 combinations). Two-tailed Mann-Whitney test. As many peptide and participant combinations showed no response, numerous points are at the limit of detection (100 SFC).

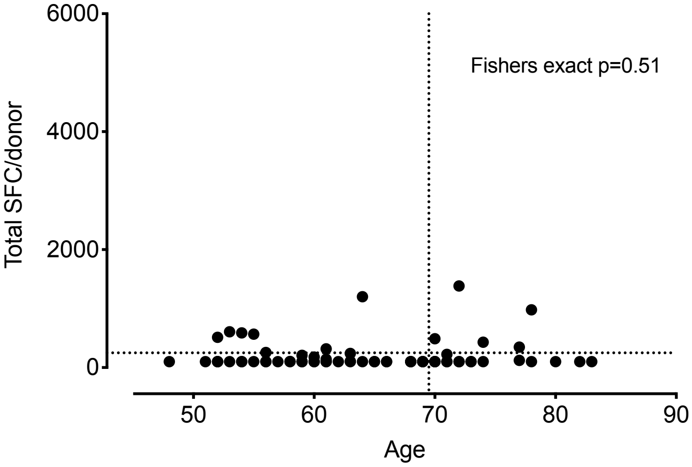

Supplementary Figure 3. Correlation between α-syn-specific T cell reactivity and age in HC. (n=67) The dotted lines indicate 250 SFC and 70 year-old cut-off for two-tailed Fisher's exact test. Total SFC is defined as the magnitude of responses (the sum of IFNγ, IL-5, and IL-10) against the α-syn peptide pool reported as SFC per 10^6 cultured PBMC.

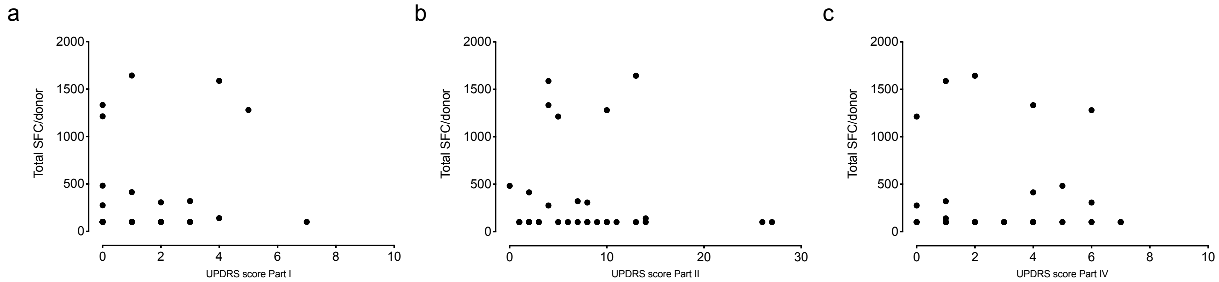

Supplementary Figure 4. Correlation between α-syn-specific T cell reactivity and UPDRS Part I, II, and IV. (a) UPDRS Part I, (b) UPDRS Part II, (c) UPDRS Part IV. (a-c) n=33. Total SFC is defined as magnitude of responses (sum of IFNγ, IL-5, and IL-10) against α-syn peptide pool as SFC per 10^6 cultured PBMC.

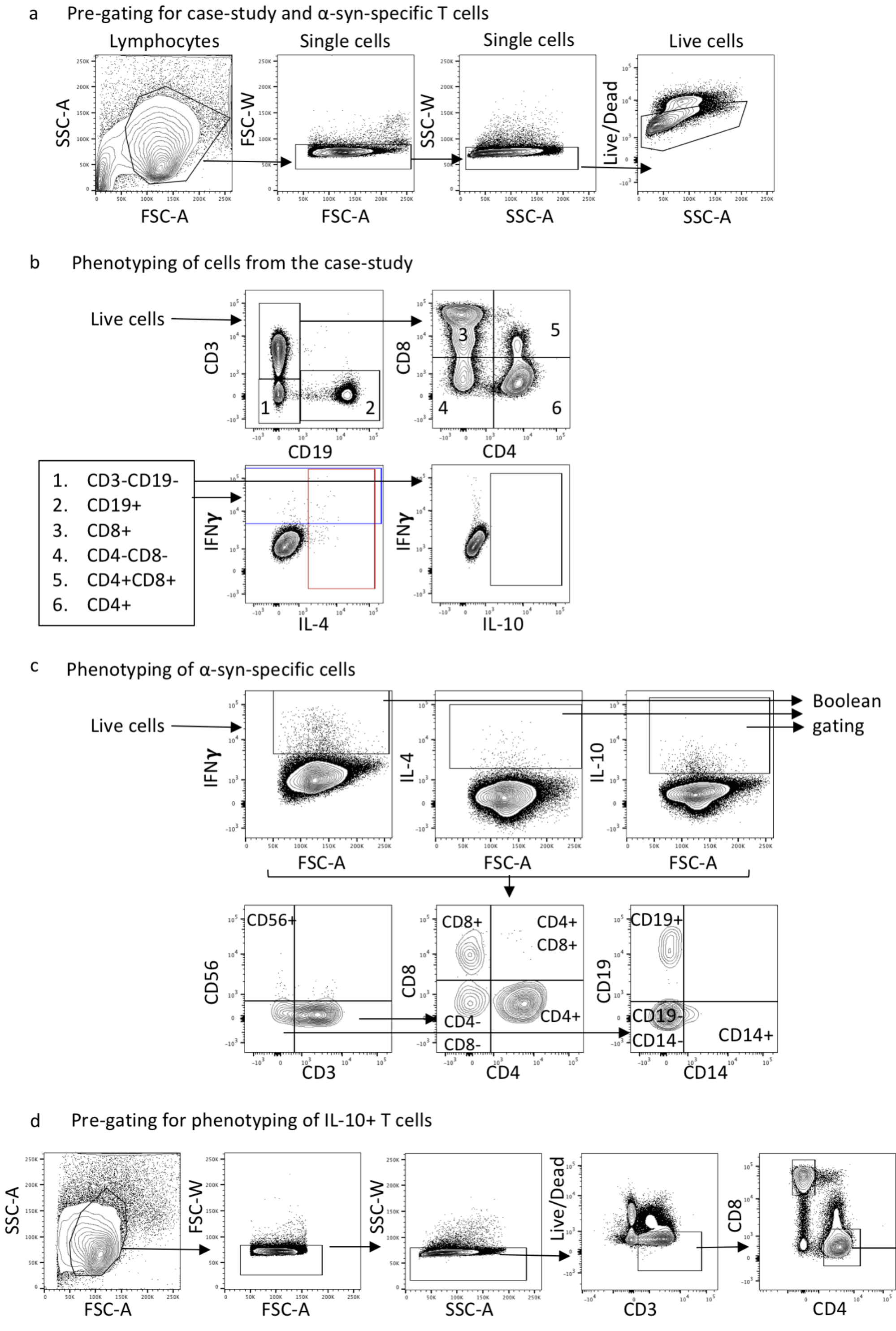

Supplementary Figure 5. Gating strategy for intracellular staining analysis. (a) Gating strategy to identify live cells from the case-study and phenotyping of α-syn-specific T cells. (b) Gating strategy to phenotype α-syn-specific cells in the case study corresponding to Fig. 1b-d. (c) Gating strategy to phenotype α-syn-specific cells corresponding to Fig. 5a-d. (d) Gating strategy to identify CD4+ T cells for phenotyping of IL-10+ T cells. Live/Dead stain also includes elimination of CD19+ and CD14+ cells. Subsequent gating is shown in Fig. 5e.

Supplementary Table 1. Lack of association between HLA alleles and PD or α-syn T cell response

Association	HLA allele	Individuals with allele		Individuals lacking allele		Relative frequency	Odds ratio	p-value[a]	Bonferroni corrected p-value
		PD	HC	PD	HC				
Disease	*DQA1*01:01*	38	14	59	53	0.66	0.41	0.017	3.3
	*C*08:02*	8	0	88	67	0.00	0.00	0.021	4.2
	*B*38:01*	8	0	88	66	0.00	0.00	0.022	4.2
	*DRB4*01:01*	40	41	53	25	1.22	2.17	0.024	4.7
	*DQB1*05:01*	29	10	65	56	0.62	0.40	0.026	5.0
	*B*14:02*	7	0	89	66	0.00	0.00	0.042	8.2
	*A*26:01*	7	0	90	64	0.00	0.00	0.043	8.3
		Resp.	Non-resp.	Resp.	Non-resp.				
α -syn response	*A*29:02*	7	3	38	113	2.50	6.94	0.005	1.1
	*DRB5*02:02*	5	2	38	114	2.64	7.50	0.016	3.1
	*DPB1*04:01*	34	67	11	52	1.23	2.40	0.031	6.0
	*DRB1*16:01*	4	2	41	117	2.43	5.71	0.049	9.5

[a] Fisher's exact test.

Supplementary Table 2. α-syn peptides used in this study

Sequence	Modification	Pool
KTKEGVLYVGSKTKE	Wt	Both
KTKEGVLXVGSKTKE	Nitrated Y	Case-study
KTKEGVLXVGSKTKE	Phosphorylated Y	Cross-sectional
DNEAYEMPSEEGYQD	Wt	Both
DNEAXEMPSEEGXQD	Nitrated Y	Both
DNEAYEMPXEEGYQD	Phosphorylated S	Both
EMPSEEGYQDYEPEA	Wt	Both
EMPSEEGXQDXEPEA	Nitrated Y	Both
EMPXEEGYQDYEPEA	Phosphorylated S	Both
MPVDPDNEAYEMPSE	Wt	Cross-sectional
MPVDPDNEAXEMPSE	Nitrated Y	Cross-sectional
MPVDPDNEAYEMPXE	Phosphorylated S	Both
EQVTNVGGAVVTGVT	Wt	Case-study
GVLYVGSKTKEGVVH	Wt	Case-study
VAEAAGKTKEGVLYV	Wt	Case-study

Table S3. Antibody panels used in flow cytometry assays

Marker	Fluorochrome	Clone	Manufacturer	Cat. No.	Staining panel[A]
CD4	APCeF780	RPA-T4	eBioscience	47004942	all
CD3	AF700	UCHT1	eBioscience	56003842	all
CD8	BV650	RPA-T8	BioLegend	301042	all
CD19	PECy7	HIB19	TONBO	600199T100	Case study, α-syn
CD19	V500	HIB19	BD Biosciences	561121	IL-10
CD14	APC	61D3	TONBO	200149T100	Case study, α-syn
CD14	V500	M5E2	BD Biosciences	561391	IL-10
CD56	PE	CMSSB	eBioscience	12056742	α-syn
CD127	PECy7	eBIORDR5	eBioscience	25127842	IL-10
CD25	PerCpCy5.5	BC96	BioLegend	302626	IL-10
IFNγ	FITC	4S.B3	eBioscience	11731982	Case study, α-syn
IL-4	PE/Dazzle594	MP4-25D2	BioLegend	500831	Case study, α-syn
IL-10	BV421	JES3-9D7	BD Biosciences	564053	α-syn
IL-10	APC	JES3-9F1	BioLegend	506807	IL-10

[A] Three different staining panels were used; case study, α-syn-specific T cell characterization (α-syn), and phenotyping of IL-10$^+$ T cells (IL-10). Antibodies used in all three panels are labelled "all".
