# Supplementary material for: α-Synuclein-specific T cell reactivity is associated with preclinical and early Parkinson’s disease
Source: Nat Commun. 2020 Apr 20;11:1875. doi: 10.1038/s41467-020-15626-w (PMC7171193; doi:10.1038/s41467-020-15626-w)
Supplement: Supplementary file 3 — Reporting Summary [file 41467_2020_15626_MOESM3_ESM.pdf]

nature research

Corresponding author(s): Alessandro Sette
David Sulzer

Last updated by author(s): Mar 13, 2020

Reporting Summary

Nature Research wishes to improve the reproducibility of the work that we publish. This form provides structure for consistency and transparency in reporting. For further information on Nature Research policies, see Authors & Referees and the Editorial Policy Checklist .

Statistics

For all statistical analyses, confirm that the following items are present in the figure legend, table legend, main text, or Methods section.

n/a	Confirmed	
☐	☒	The exact sample size (*n*) for each experimental group/condition, given as a discrete number and unit of measurement
☐	☒	A statement on whether measurements were taken from distinct samples or whether the same sample was measured repeatedly
☐	☒	The statistical test(s) used AND whether they are one- or two-sided<br>*Only common tests should be described solely by name; describe more complex techniques in the Methods section.*
☐	☒	A description of all covariates tested
☒	☐	A description of any assumptions or corrections, such as tests of normality and adjustment for multiple comparisons
☒	☐	A full description of the statistical parameters including central tendency (e.g. means) or other basic estimates (e.g. regression coefficient) AND variation (e.g. standard deviation) or associated estimates of uncertainty (e.g. confidence intervals)
☐	☒	For null hypothesis testing, the test statistic (e.g. *F*, *t*, *r*) with confidence intervals, effect sizes, degrees of freedom and *P* value noted<br>*Give P values as exact values whenever suitable.*
☒	☐	For Bayesian analysis, information on the choice of priors and Markov chain Monte Carlo settings
☒	☐	For hierarchical and complex designs, identification of the appropriate level for tests and full reporting of outcomes
☐	☒	Estimates of effect sizes (e.g. Cohen's *d*, Pearson's *r*), indicating how they were calculated

Our web collection on statistics for biologists contains articles on many of the points above.

Software and code

Policy information about availability of computer code

Data collection	No open source or custom code was used to collect the data in this study.
Data analysis	Fluorospot spot counting was performed using computer-assisted image analysis on the AID iSpot version 7 (Aid Diagnostica GMBH, Strassberg, Germany).<br>Flow cytometry data was acquired on a BD LSRII flow cytometer and analysis was done using FlowJo X Software version 10 (FlowJo LLC, Ashland, OR).<br>HLA typing was performed by an ASHI-accredited laboratory at Murdoch University. HLA alleles were determined through a proprietary allele calling algorithm and analysis pipeline (IIID HLA analysis suite; www.iiid.com.au/laboratory-testing/) using the latest IMGT HLA database as reference (www.imgt.org).<br>HLA association odds ratios and relative frequencies were calculated using the RATE program (www.iedb.org),<br>Statistical analyses was performed using GraphPad Prism version 7 and GraphPad Quickcalcs.

For manuscripts utilizing custom algorithms or software that are central to the research but not yet described in published literature, software must be made available to editors/reviewers. We strongly encourage code deposition in a community repository (e.g. GitHub). See the Nature Research guidelines for submitting code & software for further information.

Data

Policy information about availability of data

All manuscripts must include a data availability statement. This statement should provide the following information, where applicable:
- Accession codes, unique identifiers, or web links for publicly available datasets
- A list of figures that have associated raw data
- A description of any restrictions on data availability

All data generated or analyzed during this study are included in this article and its Supplementary Information. For HLA typing information from the freely available international immunogenetics information system (http://www.imgt.org), and an ASHI-accredited HLA allele caller software pipeline, IIID HLA Analysis suite (http://

www.iiid.com.au/laboratory-testing/) was used.

Field-specific reporting

Please select the one below that is the best fit for your research. If you are not sure, read the appropriate sections before making your selection.

[x] Life sciences [ ] Behavioural & social sciences [ ] Ecological, evolutionary & environmental sciences

For a reference copy of the document with all sections, see nature.com/documents/nr-reporting-summary-flat.pdf

Life sciences study design

All studies must disclose on these points even when the disclosure is negative.

Sample size	No sample size calculation was performed since we did not have an expected frequency of response. The final sample size used was based on the samples available from the recruitment sites.
Data exclusions	Data was excluded from Fluorospot analysis according to predefined criteria of either cell death in culture preventing further experiments, or a failed PHA response in the assay (<100 SFC per million cells)
Replication	The correlation between alpha-synuclein reactivity and time since diagnosis was confirmed in an independent patient cohort. Each individual sample was tested in one experiment.
Randomization	Participants were allocated into experimental groups based on PD diagnosis or HC. For cohort 1 the patients were from all recruitment sites with a spread of time since diagnosis between 0-28 years. The validation cohort were patients from UAB diagnosed 0-8 years ago. Samples studied in flow cytometry were selected based on cells available following the Fluorospot assays.<br>For the AD studies individuals were allocated into each group based on AD diagnosis or HC.<br>Assignments to each respective cohort was based on the clinical diagnosis.
Blinding	Individuals performing the experiments and collecting the raw data were blinded to cohort assignment PD vs HC or AD vs HC. Individuals analyzing the data were not blinded since the comparisons were dependent on knowing the respective cohort.

Reporting for specific materials, systems and methods

We require information from authors about some types of materials, experimental systems and methods used in many studies. Here, indicate whether each material, system or method listed is relevant to your study. If you are not sure if a list item applies to your research, read the appropriate section before selecting a response.

Materials & experimental systems

n/a	Involved in the study	
[ ]	[x]	Antibodies
[x]	[ ]	Eukaryotic cell lines
[x]	[ ]	Palaeontology
[x]	[ ]	Animals and other organisms
[ ]	[x]	Human research participants
[x]	[ ]	Clinical data

Methods

n/a	Involved in the study	
[x]	[ ]	ChIP-seq
[ ]	[x]	Flow cytometry
[x]	[ ]	MRI-based neuroimaging

Antibodies

Antibodies used	For Fluorospot antibodies from Mabtech (Sweden) was used as described in materials and methods section.<br>Mouse anti-human IFNg (clone 1-D1K), mouse anti-human IL-5 (clone TRFK5), and mouse anti-human IL-10 (clone 9D7) as capture antibodies. Secondary antibodies: IFNg-BAM (7-B6-1-FS-BAM), IL-5-WASP (5A10-WASP), and IL-10-biotin (12G8-biotin), followed by anti-BAM-490, anti-WASP-640, and Streptavidin-550.<br>For intracellular cytokine staining Table S3 lists antibodies used from eBioscience, BD Pharmingen, BioLegend, and TONBO.<br>Antibodies used for all staining panels (clone, company, catalogue number):<br>1. CD4 APCeF780 (RPA-T4, eBioscience, 47004942)<br>2. CD3 AF700 (UCHT1, eBioscience, 56003842)<br>3. CD8 BV650 (RPA-T8, BioLegend, 301042)<br>Case study and alpha-synuclein phenotyping:<br>4. CD19 PECy7 (HIB19, TONBO, 600199T100)<br>5. CD14 APC (61D3, TONBO, 200149T100)<br>6. IFNg FITC (4S.B3, eBioscience 11731982)<br>7. IL-4 PE/Dazzle594 (MP4-25D2, BioLegend, 500831)<br>IL-10 phenotyping:

8. CD19 V500 (HIB19, BD Biosciences, 561121)
9. CD14 V500 (M5E2, BD Biosciences, 561391)
10. CD127 PECy7 (eBIORDR5, eBioscience, 25127842)
11. CD25 PerCpCy5.5 (BC96, BioLegend, 302626)
12. IL-10 APC (JES3-9F1, BioLegend, 506807)
Alpha-synuclein phenotyping:
13. CD56 PE (CMSSB, eBioscience, 12056742)
14. IL-10 BV421 (JES3-9D7, BD Biosciences, 564053)

Validation

Mouse anti-human IFNg (clone 1-D1K): https://www.mabtech.com/products/anti-human-ifn-gamma-antibody-1-d1k-purified-3420-3#tabs-min-1
mouse anti-human IL-5 (clone TRFK5): https://www.mabtech.com/products/anti-human-il-5-antibody-trfk5-purified-3490-3
mouse anti-human IL-10 (clone 9D7): https://www.mabtech.com/products/anti-human-il-10-antibody-9d7-purified-3430-3
IFNg-BAM (7-B6-1-FS-BAM) followed by anti-BAM-490, IL-5-WASP (5A10-WASP) followed by anti-WASP-640, IL-10-biotin (12G8-biotin) followed by Streptavidin-550: https://www.mabtech.com/products/human-ifn-gamma-il-10-il-5-fluorospotplus-fsp-010708
1. CD4 APCeF780 (RPA-T4, eBioscience, 47004942): https://www.thermofisher.com/antibody/product/CD4-Antibody-clone-RPA-T4-Monoclonal/47-0049-42
2. CD3 AF700 (UCHT1, eBioscience, 56003842): https://www.thermofisher.com/antibody/product/CD3-Antibody-clone-UCHT1-Monoclonal/56-0038-42
3. CD8 BV650 (RPA-T8, BioLegend, 301042): https://www.biolegend.com/en-us/products/brilliant-violet-650-anti-human-cd8a-antibody-7652
4. CD19 PECy7 (HIB19, TONBO, 600199T100): https://tonbobio.com/products/pe-cyanine7-anti-human-cd19-hib19
5. CD14 APC (61D3, TONBO, 200149T100): https://tonbobio.com/products/apc-anti-human-cd14-61d3
6. IFNg FITC (4S.B3, eBioscience 11731982): https://www.thermofisher.com/antibody/product/IFN-gamma-Antibody-clone-4S-B3-Monoclonal/11-7319-82
7. IL-4 PE/Dazzle594 (MP4-25D2, BioLegend, 500831): https://www.biolegend.com/en-us/products/pe-dazzle-594-anti-human-il-4-antibody-10216
8. CD19 V500 (HIB19, BD Biosciences, 561121): https://www.bdbiosciences.com/eu/applications/research/clinical-research/oncology-research/blood-cell-disorders/surface-markers/human/v500-mouse-anti-human-cd19-hib19/p/561121
9. CD14 V500 (M5E2, BD Biosciences, 561391): https://www.bdbiosciences.com/eu/applications/research/stem-cell-research/hematopoietic-stem-cell-markers/human/negative-markers/v500-mouse-anti-human-cd14-m5e2/p/561391
10. CD127 PECy7 (eBIORDR5, eBioscience, 25127842): https://www.thermofisher.com/antibody/product/CD127-Antibody-clone-eBioRDR5-Monoclonal/25-1278-42
11. CD25 PerCpCy5.5 (BC96, BioLegend, 302626): https://www.biolegend.com/en-us/products/percpcyanine55-anti-human-cd25-antibody-4231
12. IL-10 APC (JES3-9F1, BioLegend, 506807): https://www.biolegend.com/en-us/products/apc-anti-human-il-10-antibody-1572
13. CD56 PE (CMSSB, eBioscience, 12056742): https://www.thermofisher.com/antibody/product/CD56-NCAM-Antibody-clone-CMSSB-Monoclonal/12-0567-42
14. IL-10 BV421 (JES3-9D7, BD Biosciences, 564053): https://www.bdbiosciences.com/eu/applications/research/t-cell-immunology/th-2-cells/intracellular-markers/cytokines-and-chemokines/human/bv421-rat-anti-human-and-viral-il-10-jes3-9d7/p/564053

Human research participants

Policy information about studies involving human research participants

Population characteristics	We enrolled one participant who was diagnosed with Parkinson's disease 10 years ago and who were able to provide longitudinal samples from before PD diagnosis until now.<br>As table 2 lists other participants enrolled were 97 patients with PD and 67 HC (age-matched without PD diagnosis) from RUMC, UAB and UCSD. Median age for PD was 67 and HC 64. The PD cohort consisted of 73% males and the HC cohort of 25% males. We also collected ethnicity and for PD patients age at diagnosis, years since diagnosis, UPDRS, MoCA and LED scores.<br>In table 3, 38 individuals with AD and 41 age-matched HC were enrolled from CUMC and Precision Med. Median age for AD was 69 and HC 66. The AD cohort consisted of 47% males and the HC cohort of 46% males. We also collected ethnicity, and for AD patients median age at diagnosis, years since diagnosis, MMSE and MoCA.
Recruitment	PD participants were enrolled on the basis of the following inclusion criteria: moderate to advanced PD; 2 of: rest tremor, rigidity, and/or bradykinesia, PD diagnosis at age 47-75, dopaminergic medication benefit, enrollment age 50-90, and ability to provide informed consent. The exclusion criteria were: atypical parkinsonism or other neurological disorders, history of cancer within the past 3 years (not skin), autoimmune disease (except thyroid), and chronic immune-modulatory therapy. Age-matched HC were selected on the basis of age 50-90 and ability to provide informed consent. Exclusion criteria were the same as for PD donors, and in addition we excluded self-reported genetic factors (i.e., PD in first-degree blood relative). In the LJI cohort (n=11 PD), Parkinson's disease was self-reported. These individuals were excluded from the correlations with clinical parameters, since those results were not available. Therefore, the conclusions in this manuscript are not impacted by this cohort.<br>AD subjects from CUMC were recruited according to NIA-AA criteria. They were recruited after at least 2 clinical visits. They had neuropsychological testing and in some cases positive biomarkers (SPECT scan, FDG PET scan, CSF or amyloid scan). The HC went through assessments for neurological and neuropsychological testing. They had 2 consecutive years with normal neuropsychological testing.<br>The AD cohort recruited by Precision Med were diagnosed according to NINCDS-ADRDA criteria. They underwent MRI/CT scans to rule out other causes of cognitive decline. They exhibit deficits in two or more areas of cognition and have progressive worsening of memory and other cognitive functions. The HC are self-reported without evidence for decline in cognitive functions.
Ethics oversight	All participants provided written informed consent for participation in the study. In the case of the AD cohort all the participants or their authorized representatives provided written informed consent. Ethical approval was obtained from the Institutional review boards at La Jolla Institute for Immunology (LJI; protocol numbers VD-167, VD-124, VD-118, VD-155, and VD-187), Rush University Medical Center (RUMC; Office of research affairs number 16042107-IRB01), University of California San Diego (UCSD; protocol number 161224) and University of Alabama (UAB; protocol number IRB-300001297) and CUMC (protocol number IRB-AAAQ9714).

Note that full information on the approval of the study protocol must also be provided in the manuscript.

Flow Cytometry

Plots

Confirm that:

- [x] The axis labels state the marker and fluorochrome used (e.g. CD4-FITC).
- [x] The axis scales are clearly visible. Include numbers along axes only for bottom left plot of group (a 'group' is an analysis of identical markers).
- [x] All plots are contour plots with outliers or pseudocolor plots.
- [x] A numerical value for number of cells or percentage (with statistics) is provided.

Methodology

Sample preparation	PBMCs were purified from whole blood using Ficoll-Paque density gradient centrifugation. PBMCs were then thawed and stimulated with alpha-synuclein peptide pool for 14 days. Upon harvest cells were stimulated again with alpha-synuclein epitope pool.
Instrument	BD LSR II
Software	Diva software for collection and FlowJo X for analysis.
Cell population abundance	No sorting was performed for this study.
Gating strategy	Gating strategy is defined in Supplementary figure 5.<br>Case-study: Non-lymphocytes and doublet cells were eliminated by forward and side scatter, and dead cells by Live/Dead stain. CD3+ T cells were gated based on their CD4 and CD8 expression (CD8+, CD8+CD4+, CD4+, CD8-CD4-), and CD3-CD19- and CD19+ populations. Cell populations were then gated on IFNg, IL-4, or IL-10 expression.<br>Phenotyping of alpha-synuclein specific T cells: Non-lymphocytes/monocytes and doublet cells were eliminated by forward and side scatter, and dead cells by Live/Dead stain. Cells were gated based on their cytokine expression and then cytokine-expressing cells were gated based on their CD3+ and CD56+ expression. CD3+ T cells were gated based on their CD4 and CD8 expression (CD8+, CD8+CD4+, CD4+, CD8-CD4-), and other populations based on CD3-CD56- and other markers as indicated; CD14-CD19+ (B

cells), CD14+CD19- (Monocytes), CD14-CD19- (other).
Boolean gating of cytokine-expressing cells was performed.
Phenotyping of IL-10+CD4+ T cells: Non-lymphocytes and doublet cells were eliminated by forward and side scatter, and dead cells by Live/Dead stain, as well as CD19+ and CD14+ cells. CD3+ T cells were gated based on their CD4+ expression and then on IL-10 expression. IL-10+ cells were then gated on CD25 and CD127 expression. CD4+ T cells were also gated on CD25+CD127lo followed by IL-10 expression.

☒ Tick this box to confirm that a figure exemplifying the gating strategy is provided in the Supplementary Information.
